# Prediction of Relapse Using Digital Technology in People in Recovery From Substance Use Disorders: Early Economic Evaluation With a Case Study of the Subreal App

**DOI:** 10.2196/87186

**Published:** 2026-04-14

**Authors:** Janet Bouttell, Michał Bartler, Sarah Bolton

**Affiliations:** 1 Faculty of Engineering, University of Nottingham, University Park, Nottingham, England, NG7 2RD, United Kingdom, +447809724281; 2 Subreal Limited, London, England, United Kingdom; 3 Centre for Healthcare Equipment and Technology Adoption (CHEATA), Nottingham University Hospitals NHS Trust, Nottingham, England, United Kingdom

**Keywords:** early economic evaluation, early health technology assessment, technology development, digital technology, substance use disorder, opioid use disorder, alcohol use disorder, prediction, relapse

## Abstract

**Background:**

Many people relapse after achieving abstinence in substance use disorders. Health care providers may scan the horizon for new technologies to predict response that allow interventions to be targeted rather than routine. Currently, no such predictive technologies are available in the United Kingdom. The Subreal app is available for use in research contexts, but no clinical data specific to the app are yet available. Early health economic modeling can use data from the literature to explore characteristics essential for the new technology to be cost-effective. This information can guide developers in setting performance targets and pricing and estimating potential cost savings and/or cost-effectiveness for health care providers.

**Objective:**

This study was supported by a UK industry funding body to explore the potential of digital technologies such as the Subreal app to offer cost savings or cost-effectiveness for health care providers. We explored the threshold price and clinical effectiveness required to deliver cost savings and cost-effectiveness in 2 subpopulations with substance use disorders in a UK setting.

**Methods:**

Deterministic models were used to estimate costs per relapse and quality-adjusted life years over 1-, 5-, and 20-year time horizons for people who have achieved abstinence after treatment for alcohol or opioid misuse. The intervention was a digital technology predicting relapse, provided—in addition to standard care—for 1 year post achievement of abstinence. In Subreal, biomarker data are collected daily through the app, and artificial intelligence–enhanced risk assessment flags patients who require additional support. The comparator was event-driven, reactive response to relapse. Costs and quality-of-life estimates were calculated using Markov models with data from existing published sources. The base-case estimate of 15% reduction in first-year relapse rates was based on a previous study on a similar but simpler digital technology.

**Results:**

Digital technologies such as the Subreal app have the potential to be cost-saving from a UK health and social care perspective, especially when used over a longer time horizon. Assuming a reduction of 15% in first-year relapse rates, digital technologies have the potential to be cost-saving, provided that they do not cost more than £300 (US $400.09) and £460 (US $613.47) per patient per annum for alcohol and opioid use disorders, respectively. No cost was included for postalert care, as it was assumed that this could be met within existing resources. Cost savings would be achieved predominantly through a reduction in treatment requirements as fewer people relapse. Price thresholds would reduce correspondingly if a <15% reduction in relapse rates were achieved.

**Conclusions:**

Developers of digital technologies that aim to reduce relapse need to focus on the generation of evidence of clinical effectiveness and develop a commercially sustainable pricing model that allows health care providers to benefit from cost savings.

## Introduction

### Background

Between April 2023 and March 2024, nearly 311,000 adults were in contact with drug and alcohol treatment services in England [1]. This represents a 7% rise compared with the previous year, and it is the highest number of adults in treatment since 2009-2010 [1]. Alcohol and drug use disorders cause increased morbidity and mortality and place an economic burden on the health and welfare system and potentially on the criminal legal system [2-4]. Notably, 72% and 69% of people starting treatment for alcohol use disorder (AUD) and opioid use disorder (OUD), respectively, report having mental health needs [1]. Treatment for AUD is predominantly delivered in the community, with virtually all people receiving some form of psychosocial treatment and 18% also receiving pharmacological treatment [1]. Similarly, for OUD, 99% of people on treatment receive psychosocial treatment, but rates of pharmacological treatment (94%) are higher than those for AUD [1]. People receiving treatment for AUD tend to stay in treatment for approximately 6 months; in 2023‐2024, 58% of people completed treatment and were free from dependence [1]. For OUD, people remain in treatment for much longer, with the average reported as 3.3 years in 2023‐2024 and only 23% of people completing treatment.

Substance use disorders tend to have a relapsing nature. Of the 1.2 million people who were in contact with drug and alcohol services in the United Kingdom from 2005‐2006 to 2023‐2024, 31% had more than 4 treatment journeys, 26% had been in treatment continuously since their treatment started, and 71% had been in treatment for 5 years or more [1]. A 2007 US study reported that 36% of those achieving abstinence from alcohol sustained abstinence for 12 months, whereas 86% of those sustaining abstinence for 12 months remained abstinent at 5 years [5]. For opioids, another US study, from 1986, reported that 35% relapsed immediately after treatment, whereas 73% of those who sustained abstinence for 12 months remained abstinent at 42 months [6]. These data demonstrate the importance of supporting abstinence over the first 12 months posttreatment.

The UK government has published a 10-year health plan for England, aiming to transform the National Health Service (NHS) to be fit for the future [7]. The plan focuses on embracing digital technologies and on preventing illness (including secondary prevention). Therefore, a digital technology to prevent relapse from substance use disorders is strongly aligned with UK strategic health priorities. Additionally, NHS Core20PLUS5 is a national approach to inform action to reduce inequalities at the national and system levels [8]. It targets the most deprived 20% of the national population as well as people experiencing social exclusion, specifically people with drug and alcohol dependence.

### About the Technology

The Subreal app integrates cutting-edge digital biomarkers (heart rate variability and pupillary light reflex) and artificial intelligence–enhanced predictive algorithms to enable the early detection of relapse risks, automated decision support systems for clinicians, and remote patient management that leverages real-time physiological and behavioral data. The technology is expected to drive a shift away from reactive, event-driven care to a more anticipatory and proactive continuous management approach, improving patient outcomes and reducing care costs. We anticipate that it will be useful for the care of people across a broad range of substance abuse disorders, including those who abuse a number of substances simultaneously. Predictive algorithms to detect high-relapse-risk episodes use smartphone-collected digital biomarker data and digital phenotyping techniques, including app use behavior and patterns in missing data. Physiological data are collected through the patient recording a short video and submitting it via the platform. Predictions are designed to be used as a clinical guideline for further intervention, where a clinician is notified through SMS text messaging, pager messaging, or other appropriate clinical management system about the increased relapse risk, which might incentivize patient-clinician contact and lead to the administration of a psychological or pharmacological intervention for relapse prevention. No detailed review of data by the clinician is required, as the prediction tool simply sends an alert to a designated person. The digital platform will allow for rapid scalability across different clinical settings, allowing for widespread uptake and improvement in patient care. The benefits are expected to be realized through reducing relapse rates, hospital admissions, and overall health care burden.

**Figure 1.**
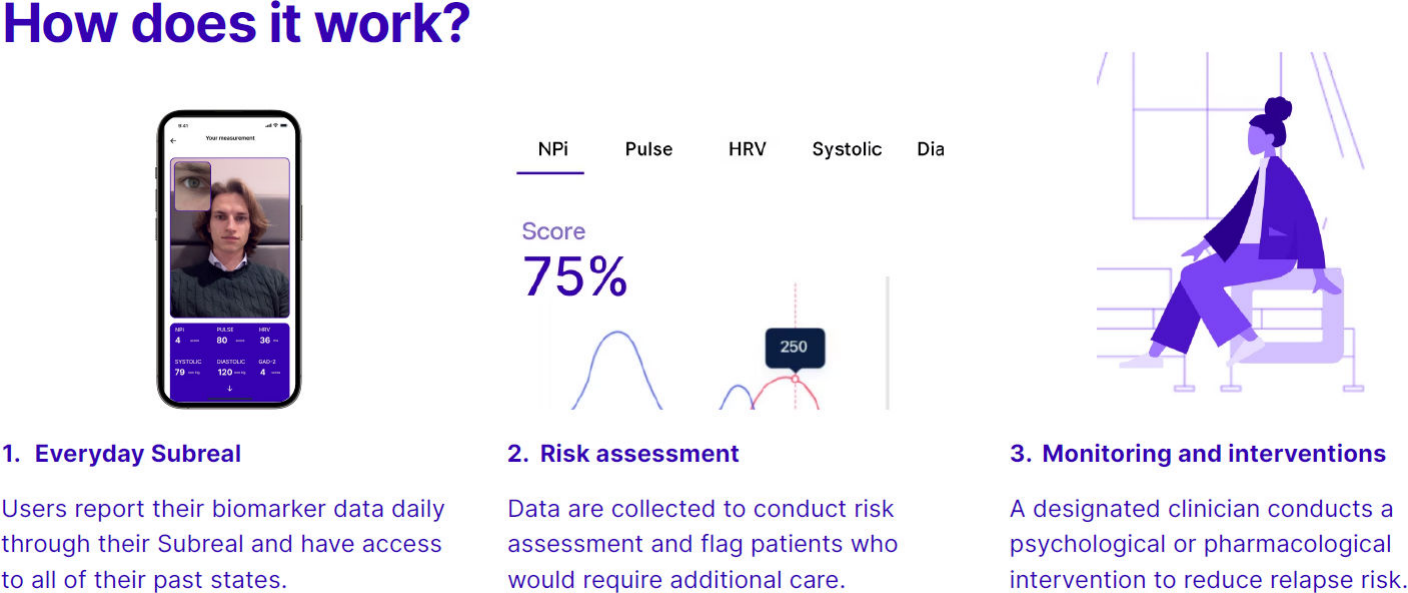
Subreal app—the proposed clinical pathway.

The app is currently at a preclinical stage (Technology Readiness Level 4 [TRL 4]) as an investigational medical device under the European Union medical device regulations. Similar predictive solutions for alcohol and opioids already exist on the wider European market, including Kontigo Care Previct Alcohol and Previct Drugs operating in Sweden in clinical practice. Subreal differs from Previct, as it uses physiological data captured via smartphone (Figure 1), whereas Previct uses a combination of breathalyzer data and self-reported patient data from questionnaires and journals [9].

### Early Exploratory Economic Evaluation

Economic evaluation is the comparative analysis of the costs and consequences of 2 or more possible options [10]. It is undertaken as part of technology adoption decision-making at many health technology assessment (HTA) bodies around the world. In the United Kingdom, cost-utility analysis is undertaken by the National Institute of Health and Care Excellence (NICE) to inform decisions about whether or not to adopt a technology in a given indication at a fixed price [11]. The economic evaluation approach used by NICE can be adapted to inform developers as part of what has become known as “early HTA” [11]. By definition, this analysis may be undertaken before clinical effectiveness evidence specific to the technology is available. The purpose of early HTA is to inform developers of health technologies and their funders and investors about the potential value of their technology in a particular context and to explore what characteristics are essential to realize that value [12,13]. Early HTA identifies target clinical effectiveness and price levels that would be acceptable to health care providers [13]. Publication of early analyses can also be useful for health care providers in horizon scanning for technologies to meet a clinical need and in deciding whether to support research efforts related to technologies in development. Research funders such as the National Institute for Health Research are increasingly asking for an early health economic evaluation to be undertaken as a requirement for funding.

### Objectives of the Study

The objective of this study was to provide an estimate of potential cost-effectiveness or cost saving of introducing a digital technology that enables early detection of relapses (the Subreal app) to the clinical pathway in the first year of recovery for people with AUD or OUD. This will inform developers and investors about pricing policy and necessary performance of the technology at different price levels. It will also inform potential adopters and clinical research partners about the potential cost and health outcome implications of adopting a technology of this type.

## Methods

### Decision Problem

The first stage of an economic evaluation is to determine the scope of the decision problem. This is often done using the Population, Intervention, Comparator, and Outcome (PICO) framework. The PICO framework for this study is presented in Textbox 1.

Textbox 1. **Population, Intervention, Comparator, and Outcome (PICO) for scope of the economic evaluation.**

- Population (P):

1. people with moderate or severe alcohol use disorder who have successfully completed treatment but remain at risk of relapse
2. people with opioid use disorder who have successfully completed treatment but remain at risk of relapse

- Intervention (I): standard-of-care pathway including a digital technology, such as the Subreal app, for the first year of abstinence
- Comparator (C): standard of care
- Outcome (O): quality-adjusted life years (QALYs), costs per patient

People who have completed treatment and achieved abstinence for AUD and OUD were considered as 2 separate populations. These populations were chosen, as they are the largest patient groups in the UK substance abuse statistics—representing 44% and 30% of adults in treatment, respectively—in 2023‐2024 [1]. In both cases, the intervention was the provision of the Subreal app for the first year of abstinence. In addition, health care providers would need to design some process to build the app into their standard of care (ie, monitoring and responding to alerts and reducing routine check-ins). As this was a preliminary economic evaluation and there could be significant variation in the approach taken by health care providers, we assumed that any changes in process could be achieved using existing resources. At a later stage in development, pathways including the Subreal app will be codeveloped with clinical partners. The comparator was usual treatment (also referred to as standard of care). The outcomes of interest for a cost-utility analysis were the costs and quality-adjusted life years (QALYs) associated with the full pathway of care. QALYs are a generic measure combining quality and length of life, where 1 year in perfect health is equivalent to 1 QALY [14]. This is a useful measure, as it allows interventions in different disease areas to be compared for value for money using a common denominator. We also included cost per relapse avoided, as it may be a useful indicator for health service decision makers.

### Analytic Approach

The analysis was a cost-utility analysis comparing costs and QALYs. Costs per relapse were also calculated. A currency exchange rate of £1=US $1.33 is applicable. The estimates were used not only to estimate the potential impact on costs and QALYs separately but also to calculate the net monetary benefit (NMB). The NMB was calculated by multiplying the impact on QALYs by the UK willingness-to-pay threshold per QALY and then adjusting for the cost impact. Currently, the UK willingness-to-pay threshold per QALY is between £20,000 and £30,000; we used £20,000 in our calculations [15]. A positive NMB indicates that a technology is cost-effective. Markov models were used to model the impact of Subreal over 1-, 5-, and 20-year time horizons. This model structure is useful for relapsing conditions, as it permits people to move between disease, treatment, and disease-free states multiple times. The 1- and 5-year time horizons were selected because health care providers often want to understand whether a technology will deliver cost savings and/or be cost-effective in the short and medium term. The 20-year time horizon was included because both costs and health outcomes may continue to be impacted over a person’s lifetime. Costs and outcomes were discounted at 3.5% per annum, in line with the NICE reference case [15]. A UK health and social care perspective was adopted, which means that only costs incurred by the health and social care systems were taken into account. This is a standard approach in the assessment of UK health technologies [15]. Impacts of reducing relapses may also reduce wider costs to society (eg, in the criminal justice system or welfare payments such as unemployment benefits). There may also be costs to the person such as out-of-pocket expenses for private care or loss of earnings due to treatment or morbidity. Including these costs is known as taking a societal perspective. There may be an opportunity during a subsequent clinical study to collect these cost data and present a societal perspective as a sensitivity analysis together with the health and social care perspective.

### Model Structure

**Figure 2.**
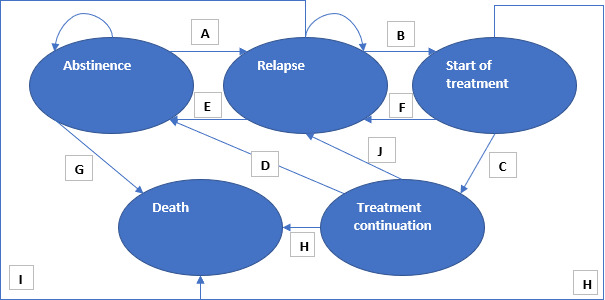
Structure of the alcohol Markov model. Letters A-J represent transition probabilities. A, relapse rate; B, treatment-seeking rate; C, treatment continuation rate; D, recovery rate on completion of treatment; E, spontaneous recovery rate; F, dropout rate (1 – treatment continuation rate); G, mortality rate from abstinence; H, mortality rate from treatment; I, mortality rate from relapse; J, nonrecovery rate (1 – recovery rate on completion of treatment). Death is an absorbing state. People can remain in abstinence and relapse states.

Figures2  3 show the structure of the Markov models used in this study. People in the alcohol model start in the abstinence state. Death is possible from any health state. They can remain abstinent or relapse. From the relapse state, people can spontaneously recover, start treatment, or remain in the relapse state. From the treatment start state, people can continue their treatment or drop out. From treatment continuation, people can return to the abstinence state or relapse. The cycle length was set to 3 months, as it allows for some people to drop out of treatment after 3 months and for the start and continuation of treatment to roughly approximate the average length of treatment for AUD (6 months).

The opioid model was similar to the alcohol model, except that the treatment start and treatment continuation were renamed medically supervised withdrawal and medication for opioid use disorder (MOUD), respectively. People can complete medically supervised withdrawal and then relapse or can continue to MOUD. People cannot relapse from MOUD, but they can remain in MOUD indefinitely. Again, the cycles were 3 months long, reflecting that medically supervised withdrawal is generally approximately 12 weeks long and that MOUD can continue for long periods.

Both models were set up to allow for the Subreal app to impact relapse rates, treatment-seeking rates, and recovery rates. No impact was included in the base case for treatment-seeking rates and recovery rates. However, if it were to become apparent during evidence generation that there was impact in these areas, it could be incorporated at a later date.

**Figure 3.**
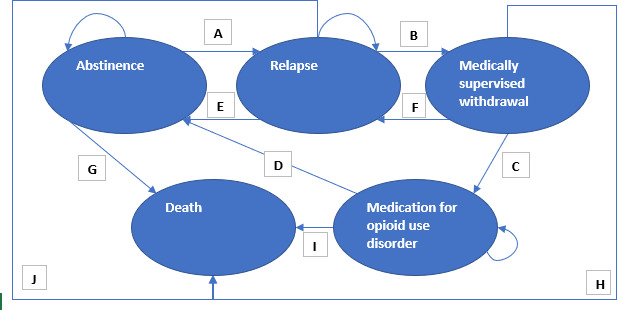
Structure of the opioid Markov model. Letters A-J represent transition probabilities. A, relapse rate; B, treatment-seeking rate; C, treatment continuation rate; D, recovery rate on completion of treatment; E, spontaneous recovery rate; F, dropout rate (1 – treatment continuation rate); G, mortality rate from abstinence; H, mortality rate from medically supervised withdrawal; I, mortality rate from medication for opioid use disorder (MOUD); J, mortality rate from relapse. Death is an absorbing state. People can remain in abstinence, relapse, and MOUD states.

### Parameter Estimates

Tables1  2 contain details of parameter estimates used to populate the models. The key parameters for the cost-effectiveness of any new health technology are the incremental cost of the technology and its clinical effectiveness. No clinical effectiveness data specific to the Subreal app are available, as this is an early economic evaluation. We assumed that the clinical effectiveness of the digital health technology is 15% in the base case, in line with that achieved in a published clinical study [9]. This was varied across a wide range in threshold analysis. We included £25 per 3-month cycle or £100 per annum for the incremental cost of the digital technology. This was based on an assumption and was varied in threshold analysis. For some parameters, the data provided in referenced papers were manipulated. Supporting calculations and comments on sources for these estimates are included in Multimedia Appendix 1.

**Table 1.** Parameter estimates for the alcohol model (the base-case estimate and lower and upper limits for 1-way sensitivity analysis).

Parameter	Estimate	Lower limit	Upper limit	Source
Transition probability				
Relapse rate – year 1	0.13	0.11	0.16	Dennis et al [5]
Relapse rate – years 2‐3	0.08	0.06	0.09	Dennis et al [5]
Relapse rate – year 4 onwards	0.03	0.03	0.04	Dennis et al [5]
Treatment-seeking rate	0.8	0.64	0.8	Assumption
Recovery rate after treatment	1	0.8	1	Assumption
Treatment continuation rate	0.58	0.46	0.7	Adult substance misuse treatment statistics 2003-2004 [1]
Spontaneous recovery rate	0.002	0.001	0.002	Mellor et al [16]
Relative mortality rate for abstainer vs occasional drinker	1.04	0.94	1.16	Zhao et al [17]
Relative mortality rate for high-volume drinker vs occasional drinker	1.24	1.07	1.44	Zhao et al [17]
Impact of Subreal app				
Impact on relapse rate	0.85	0.68	1.02	Wallden et al [9]
Impact on treatment-seeking rate	1	0.8	1.2	Assumption
Impact on recovery rate	1	0.8	1.2	Assumption
Costs^a^, GBP^b^				
Cost in abstinence state	0	0	0	Assumption
Cost in treatment states	2086	1668	2503	See Multimedia Appendix 2
Cost in relapse state	1223	979	1468	See Multimedia Appendix 2
Cost of Subreal app (per patient per 3-month cycle for 1 year)	25	25	25	Assumption
Quality of life				
Utility in abstinence state (full-year utility)	0.67	0.53	0.8	UKATT^c^ trial [18]
Utility in treatment (full-year utility)	0.62	0.5	0.74	UKATT trial [18]
Utility in relapse state (full-year utility)	0.57	0.46	0.69	UKATT trial [18]
Discount rates				
Discount rate for costs	0.035	0.01	0.05	NICE reference case [15]
Discount rate for outcomes	0.035	0.01	0.05	NICE reference case [15]
Proportion of male individuals in the population	0.74	0.59	0.89	UKATT trial [18]

a A currency exchange rate of £1=US $1.33 is applicable.

b GBP: Pound Sterling.

c UKATT: UK alcohol treatment trial.

**Table 2.** Parameter estimates for opioid model (the base-case estimate and lower and upper limits for 1-way sensitivity analysis).

Parameter	Estimate	Lower limit	Upper limit	Source
Transition probability				
Relapse rate – cycle 1	0.35	0.28	0.42	Simpson and Marsh [6]
Relapse rate – cycles 2‐4	0.06	0.05	0.08	Simpson and Marsh [6]
Relapse rate – cycles 5‐8	0.04	0.03	0.05	Simpson and Marsh [6]
Relapse rate – cycle 9 onwards	0.02	0.02	0.02	Simpson and Marsh [6]
Treatment-seeking rate	0.9	0.72	0.8	Assumption
Recovery rate after treatment	0.02	0.01	0.02	Adult substance misuse treatment statistics 2023-2024 [1]
Treatment continuation rate	1	0.8	1	Assumption
Spontaneous recovery rate	0	0	0	Assumption
Relative mortality rate for medically supervised withdrawal state	6.04	4.83	7.25	Kelty et al [19]
Relative mortality rate for MOUD state	3.55	2.84	4.26	Kelty et al [19]
Relative mortality rate for relapse state (off treatment)	6.14	4.91	7.36	Kelty et al [19]
Impact of Subreal app				
Impact on relapse rate	0.85	0.68	1.02	Wallden et al [9]
Impact on treatment-seeking rate	1	0.8	1.2	Assumption
Impact on recovery rate	1	0.8	1.2	Assumption
Costs^a^, GBP^b^				
Cost in abstinence state	0	0	0	Assumption
Cost in treatment states	2324	1959	2759	See Multimedia Appendix 2
Cost in relapse state	0	0	0	See Multimedia Appendix 2
Cost of Subreal app (per patient per 3-month cycle for 1 year)	25	25	25	Assumption—not varied in OWSA^d^, varied in threshold analysis
Quality of life				
Utility in abstinence state (full-year utility)	0.9	0.72	1	UK population norm (Janssen et al [20])
Utility in treatment (full-year utility)	0.62	0.5	0.74	NICE^c^ TAG^e^115 [21]
Utility in relapse state (full-year utility)	0.57	0.46	0.69	NICE TAG115 [21]
Discount rates				
Discount rate for costs	0.035	0.01	0.05	NICE reference case [15]
Discount rate for outcomes	0.035	0.01	0.05	NICE reference case [15]
Proportion of male individuals in the population	0.73	0.58	0.87	Adult substance misuse treatment statistics 2023-2024 [1]

a A currency exchange rate of £1=US $1.33 is applicable.

b GBP: Pound Sterling.

c NICE: National Institute for Health and Care Excellence.

d OWSA: 1-way sensitivity analysis.

e TAG: technology appraisal guidance.

### Dealing With Uncertainty

A deterministic approach was taken in the 2 models. This means that a single estimate was used for each parameter to populate the model. The NICE reference case [15] specifies that a probabilistic approach should be taken in a cost-utility analysis. Probabilistic sensitivity analysis uses a probability distribution for each parameter, which more accurately reflects the joint uncertainty in the cost and quality-of-life outcomes. This approach is believed to be inappropriate for an early exploratory health economic model, as it can lead to an impression of spurious accuracy when key clinical effectiveness data are missing [13]. This approach will be more appropriate following a clinical trial of the Subreal app. For this study, we used 1-way sensitivity analysis, which varies a single parameter at a time, thus showing the importance of individual estimates for the overall result. One-way sensitivity analysis varies parameters up and down by 20%. We also performed some specific threshold analyses to determine what the price needs to be at given effect sizes and what the effect size needs to be at given price levels. Due to data limitations, no subgroup analysis or characterization of distributional effects was planned.

### Validation

The internal validity of the models was checked by using extreme values and review by a second health economist. Face validity was confirmed by clinician review and comparison to other long-term sources (eg, for long-term relapse rates and mortality). No other stakeholders were involved in the planning or validation of the study due to resource and time constraints.

### Reporting

We used the revised CHEERS (Consolidated Health Economic Evaluation Reporting Standards) checklist [22] to ensure that our reporting was comprehensive. A completed questionnaire is provided as Checklist 1.

### Preregistration

Our study protocol was not preregistered due to the exploratory nature of the analysis. For the same reason, no health economic analysis plan was developed.

### Ethical Considerations

No ethics approval was needed for this study, as it relied solely on data from the published literature or other publicly available sources. No confidential patient data were used. The individual shown in Figure 1 provided consent for his image to be used.

## Results

### Base-Case Results

Tables3  4 present the base-case results for the alcohol and opioid models, respectively.

The alcohol model estimated very small QALY gains over 1-, 5-, and 20-year time horizons and cost savings of £129, £256, and £256, respectively (Table 3). This resulted in an NMB of £163, £299, and £300, respectively. The majority of the benefit came from cost savings due to reduced cycles of treatment, as the number of relapses was lower with the use of the Subreal app.The savings made for each AUD relapse avoided were £1642 in the first year and higher in subsequent years, as the long-term implications of a relapse avoided were also included.

Similarly, the opioid model estimated very small QALY gains over 1-, 5-, and 20-year time horizons but larger cost savings of £261, £2747, and £2750, respectively (Table 4). This resulted in an NMB of £397, £3529, and £3757, respectively. Again, the majority of the benefit came from cost savings due to reduced cycles of treatment, as the number of relapses was lower with the use of the Subreal app. The NMB value in opioid model was higher than that in the alcohol model because OUD relapses are more frequent and more costly given long-term treatment and low recovery rates. The savings made for each OUD relapse avoided were £3884 in the first year and significantly higher in subsequent years, as the long-term implications of a relapse avoided were also included.

**Table 3.** Base-case results for the alcohol model.

Alcohol model	Relapses	Savings per relapse avoided^a^, GBP^b^	QALYs^c^	Costs^a^, GBP	NMB^d^, ^a^GBP
1-year time horizon					
SOC^e^ plus Subreal app	0.53	—^f^	0.6310	1561	—
SOC	0.61	—	0.6292	1690	—
Difference per patient	−0.08	1642	0.0017	−129	163
NMB for population^g^	—	—	—	—	6.2 m^h^
5-year time horizon					
SOC plus Subreal app	5.63	—	9.1316	16,075	—
SOC	5.72	—	9.1294	16,330	—
Difference per patient	−0.10	2669	0.002	−256	299
NMB for population	—	—	—	—	11.3 m
20-year time horizon					
SOC plus Subreal app	8.91	—	12.444	20,837	—
SOC	9.00	—	12.442	21,092	—
Difference per patient	−0.10	2669	0.002	−256	300
NMB for population	—	—	—	—	11.3 m

a A currency exchange rate of £1=US $1.33 is applicable.

b GBP: Pound Sterling.

c QALYs: quality-adjusted life years.

d NMB: net monetary benefit.

e SOC: standard of care.

f Not applicable.

g Population is % people successfully completing treatment multiplied by the number of people starting treatment, from the adult substance misuse treatment statistics 2023-2024 [1].

h m: million.

**Table 4.** Base-case results for the opioid model.

Opioid model	Relapses	Savings per relapse avoided^a^, GBP^b^	QALYs^c^	Costs^a^, GBP	NMB^d^, ^a^GBP
1-year time horizon					
SOC^e^ plus Subreal app	0.44	—^f^	0.8252	2240	—
SOC	0.51	—	0.8183	2501	—
Difference per patient	−0.07	3884	0.0068	−261	397
NMB for population^g^	—	—	—	—	1.3 m^h^
5-year time horizon					
SOC plus Subreal app	1.24	—	11.6571	59,104	—
SOC	1.27	—	11.6180	61,851	—
Difference per patient	−0.03	86,633	0.0391	−2747	3529
NMB for population	—	—	—	—	11.7 m
20-year time horizon					
SOC plus Subreal app	1.64	—	14.7428	74,027	—
SOC	1.67	—	14.6924	76,777	—
Difference per patient	−0.03	94,550	0.0504	−2750	3757
NMB for population	—	—	—	—	12.5 m

a A currency exchange rate of £1=US $1.33 is applicable.

b GBP: Pound Sterling.

c QALYs: quality-adjusted life years.

d NMB: net monetary benefit.

e SOC: standard of care.

f Not applicable.

g Population is % people successfully completing treatment multiplied by the number of people starting treatment, from the Adult substance misuse treatment statistics 2023-2024 [1].

h m: million.

### Threshold Analysis

Tables5  6 show the results of a 2-way threshold analysis, which explored the effects of various levels of impact on relapse rates alongside the effect of different price levels for the Subreal app. Table 5 shows that in a population with AUD, at £100 per annum (base case), the Subreal app would be cost-effective over a 1-year time horizon with a reduction in relapse rates of 10%. At £250 per annum, the reduction in relapses would need to be 15%, and at £350 per annum, it would need to be 20%. Table 6 shows that in a population with OUD, at £100 per annum (base case), the Subreal app is likely to be cost-effective if it achieves any reduction in relapse rates. At a price of £350 per annum, a reduction in relapse rates of 10%-15% is required for the Subreal app to be cost-effective. At a price of £700 per annum, the reduction in relapse rates would have to be greater than 20% for the app to be cost-effective.

**Table 5.** Two-way threshold analysis of the effects of incremental cost and impact of the Subreal app on relapse rates on the net monetary benefit over a 1-year time horizon (all values in GBP)^a^ in a population with alcohol use disorder.

Incremental cost of Subreal app per patient per annum	£100	£250	£350	£700
5% reduction in relapse rates	−11^c^	−156	−253	−591
10% reduction in relapse rates	76	−69	−166	−504
15% reduction in relapse rates	163^b^	19	−78	−416
20% reduction in relapse rates	253	108	11	−327

a A currency exchange rate of £1=US $1.33 is applicable.

b Base-case value.

c A negative net monetary benefit indicates that the app is not cost-effective.

**Table 6.** Two-way threshold analysis of the effects of incremental cost and impact of the Subreal app on relapse rates on the net monetary benefit over a 1-year time horizon (all values in GBP)^a^ in a population with opioid use disorder.

Incremental cost of Subreal app per patient per annum	£100	£250	£350	£700
5% reduction in relapse rates	67	−78^c^	−175	−513
10% reduction in relapse rates	231	86	−10	−349
15% reduction in relapse rates	397^b^	252	155	−183
20% reduction in relapse rates	564	419	322	−16

a A currency exchange rate of £1=US $1.33 is applicable.

b Base-case value.

c A negative net monetary benefit indicates that the app is not cost-effective.

### One-Way Sensitivity Analysis

MultimediaAppendices 2  3 illustrate the results of the 1-way sensitivity analysis for the alcohol and opioid models, respectively.

The most influential parameter was the impact of the digital technology on the relapse rate. This was the only parameter that had the potential to eliminate the base-case NMB when reduced by 20%. Most other parameters had limited impact, as they influenced both the standard of care pathway and the pathway including the digital technology. The cost of implementing the digital technology would also be highly influential; however, the cost of the technology was not included in this analysis, as it was explored in the 2-way threshold analysis.

As for the alcohol model, the most influential parameter in the opioid model was the impact of the digital technology on the relapse rate. The other parameters had less impact, as they affected the results for both treatment pathways. The cost of the technology is also influential and was explored in the 2-way threshold analysis.

## Discussion

### Findings of This Study

The early health economic models demonstrated that a digital health technology predicting relapse, such as the Subreal app, has the potential to be cost-effective and cost saving as part of the care pathways for people with AUD or OUD in the United Kingdom. The study identified the combinations of the impact on relapse rates and annual fees per patient that result in a technology that is cost-effective from a UK health and social care perspective. The model results indicate that the developers should focus on generating evidence on the impact of the Subreal app on relapse rates, as this was the parameter that most influenced the results of the health economic analysis. The study indicates that the potential for cost-effectiveness and cost saving increases as longer time horizons are explored but that there is potential for cost saving within a 1-year time horizon.

### Comparison With Previous Economic Evaluations

We are not aware of any other economic evaluations of technologies to predict relapse in people with substance use disorder who have achieved abstinence. Economic evaluations in the area of AUD or OUD take a variety of analytical approaches and use different perspectives and time horizons. It is difficult to directly compare the results of these analyses with this study. However, they are useful in describing the range of costs that may be appropriate, with some studies including the costs to the criminal justice system [18,23,24]. Some studies have also provided quality-of-life estimates, which were useful to benchmark the utilities included in the models.

### Strengths and Limitations

The models presented allow the developers of a digital technology designed to reduce relapse rates to explore the size of clinical effect that may be required to support different price levels. We are not aware of another decision model that has been designed to facilitate this. The models are available to other developers from the corresponding author upon request. The models have several limitations mainly arising from either a lack of available data in the literature or restrictions in the literature search due to resource constraints that are typical in early models [24]. We performed targeted literature searches to populate the models, rather than conducting any systematic reviews. Some of the data we used, particularly for relapse rates, are from the United States and are quite dated. Other sources were not ideally suited to our purpose; for example, the UK alcohol treatment trial (UKATT) [18] costs and quality-of-life estimates are for mixed groups of abstainers and individuals who continue to consume alcohol. We have also made a number of assumptions such as the proportion of people who would seek treatment after relapsing. The limitation of data sources is a frequent problem in early health economic modeling, where the methods adopted need to be tailored to the resources available for the analysis. This generally precludes significant data analysis elements or full-scale systematic reviews. However, as the sensitivity analysis demonstrated, the only influential parameters are the price of the technology and its clinical effectiveness. All other parameters are unlikely to influence the overall result because they impact both sides of the models. The models explored only 1 position in the AUD and OUD pathways, that is, people who have recently achieved abstinence. It is possible that the findingsare generalizable to other positions in the pathway where there is potential to increase support in order to prevent an adverse outcome (such as dropping out of treatment or relapsing while on MOUD). Clinical evidence would be required to support the size of the reduction in adverse events, but the economic argument is essentially the same. The models considered only AUD and OUD, but the population potentially served by the digital technology may be broader. For example, users of the Subreal app may have a combination of different substance use disorders—that is, polysubstance use disorder—which was not accounted for in the models. The key findings of the models would likely hold true regardless of the type of substance use disorder, provided that a reduction in relapses could be demonstrated in clinical studies. We adopted a health and social care perspective, although it is known that substance use disorders have wider societal costs. These wider costs could be considered at a later development stage, and their inclusion would likely increase the probability of cost-effectiveness, as relapse would have a higher associated cost. Although our analysis considered UK data, the results are likely to be generalizable in any health system that provides a similar level of care to people with substance use disorders.

### Implications and Recommendations

The study suggests that digital technologies predicting relapse in people with AUD and OUD could be cost saving and cost-effective. Such technologies offer a potential commercial opportunity for developers, provided that the technology could be delivered for a cost, which would also allow for some of the cost to be retained by health care providers. It is vital that developers of digital technologies predicting relapse generate evidence of clinical effectiveness compared to standard of care in different populations of people who have achieved abstinence following treatment for substance use disorders. It should be noted that abstinence is not a goal for many people in treatment for OUD and the developers may wish to explore whether and how a digital technology could be useful as part of their care pathway. Health care providers may wish to consider whether this technology could be incorporated into care pathways, as it may offer cost savings over short and long time frames, as well as improving patient health (and therefore wider societal) outcomes. Practical problems to be resolved in collaboration with health care providers include how technology could be provided to allow equitable access and process redesign to incorporate the use of the app.

## Supplementary material

10.2196/87186 Multimedia Appendix 1 Additional detail on parameter estimates.

10.2196/87186 Multimedia Appendix 2 Tornado diagram illustrating the results of the 1-way sensitivity analysis for the alcohol model (blue bars depict the lower limit, while red bars depict the higher limit). NMB, net monetary benefit.

10.2196/87186 Multimedia Appendix 3 Tornado diagram illustrating the results of the 1-way sensitivity analysis for the opioid model (blue bars depict the lower limit, while red bars depict the higher limit). MSW, medically supervised withdrawal; MOUD, medication for opioid use disorder; NMB, net monetary benefit.

10.2196/87186 Checklist 1 CHEERS checklist.

## Abbreviations

AUD: alcohol use disorder
CHEERS: Consolidated Health Economic Evaluation Reporting Standards
HTA: health technology assessment
MOUD: medication for opioid use disorder
NHS: National Health Service
NICE: National Institute for Health and Care Excellence
NMB: net monetary benefit
OUD: opioid use disorder
PICO: Population, Intervention, Comparator, and Outcome
QALYs: quality-adjusted life years
TRL: Technology Readiness Level
UKATT: UK alcohol treatment trial
