# Supplementary material for: Prediction of Relapse Using Digital Technology in People in Recovery From Substance Use Disorders: Early Economic Evaluation With a Case Study of the Subreal App
Source: JMIR Form Res. 2026 Apr 14;10:e87186. doi: 10.2196/87186 (PMC13078403; doi:10.2196/87186)
Supplement: Multimedia Appendix 1 [file formative-v10-e87186-s001.pdf]

MMA2 - Costs and health outcomes parameter estimates – additional detail on sources and calculations

Reference numbers refer to those from the main manuscript

Alcohol model

Transition probabilities

Transition probabilities are the proportions of people who move between each health state each 3-month cycle. The alcohol relapse rate is based on data from Dennis et al, 2007 [13] which is a US based cohort study with a relatively high proportion of African American people and a high level of homelessness. This data is not necessarily representative of the UK population but has been used in the absence of better data. The impact of these figures on results is tested in sensitivity analysis. The paper suggests that 64% of those abstaining for less than a year will relapse, 34% of those abstaining for 12 months will relapse before 3 years and 14% of those abstaining for 3 years will relapse thereafter. These translate to three-month relapse rates of 13% for the first year, 8% for years 2-3 and 3% thereafter.

An assumption has been made that 80% of patients who relapse seek treatment. The probabilities of patients completing and succeeding at treatment are taken from national UK statistics for alcohol misuse (excluding those who misuse both drugs and alcohol) [1]. The spontaneous recovery rate is based on Mellor et al, 2021 [14]. This Australian study used survey evidence from former drinkers to estimate the proportion who recovered without treatment. Mortality rates are adjusted from sex-adjusted England population norms using relative rates from Zhao et al, 2023 [15], a systematic review and meta-analysis combining data from 107 studies. People in the abstinent and treatment states have a mortality rate 1.04 higher than occasional drinkers. People in the relapsed state are assumed to have a relative rate equivalent to a high-volume drinker (45<65 grams of ethanol per day, where 8g represents a unit of alcohol) of 1.24 compared to occasional drinkers.

Impact of Subreal App (both models)

The models are set up so that Subreal App can impact the model by reducing relapse rates, improving rates of treatment-seeking after relapse and by improving recovery after treatment. In the base case the only impact of Subreal App included is in reducing relapse rates as this is a plausible impact of a technology which gives prior warning of likely relapse allowing intervention. Other impacts could be included if a plausible clinical case could be made. In the base case it is assumed that Subreal App reduces relapses by 15% in line with evidence from Wallden et al, 2024 [16] that a 9% reduction in short term relapses and 18% reduction in longer relapses could be achieved by patients using the Previct app which is an AI-based relapse prediction tool

Costs

Costs are based on a tool for NHS commissioners using 2016-17 costs (see Figure SM1).

Figure SMI - extract from commissioning tool Alcohol-and-drugs-treatment-commissioning_tool-guidance-document.pdf)

Table 1: Unit costs (2016-17prices)

Intervention/ setting	Spend per day (drugs)	Spend per day (alcohol)
Pharmacological	£8.17	£6.27
Psychosocial	£10.19	£14.53
Inpatient treatment	£164.72	£164.72
Residential treatment	£103.57	£103.57

On rechecking the link at 22 July 2025 on this tool is no longer available and future versions are restricted to commissioners. In the absence of better data, we decided to continue to use these base costs (adjusted for time) but later models may be able to find an updated source. Table 1 shows the daily costs from Figure 1, uplifted to 2023-2024 values using the CPI index for Health from the Office for National Statistics (available at CPI INDEX 06 : HEALTH 2015=100 - Office for National Statistics). The proportion of people having each type of treatment is taken from the latest data available at Adult substance misuse treatment statistics 2023 to 2024: report - GOV.UK. 199 days of pharmacological and psychosocial treatment is based on the same report where on average people having treatment for alcohol problems completed treatment in 199 days. 14 days in-patient treatment and 30 days residential treatment are based on assumptions.

Table SM1 - updating and weighting alcohol costs (GBP)

Element of treatment – cost per day	2016-2017 (see Fig 1)	2023-2024	Days of treatment	Proportion of people	Total cost
Pharmacological	6.27	8.00	199	0.18	286.44
Psychosocial	14.53	18.53	199	1	3,687.67
In-patient	164.72	210.08	14	0.04	117.64
Residential	103.57	132.09	30	0.02	79.25
Per 6-month treatment					**4,171.00**
Per 3-month cycle					**2,085.50**

In the alcohol model costs attached to the relapsed state are taken from the UKATT RCT, 2005 uplifted to 2024 [17] using the CPI index (see link above). The UKATT trial was a UK-based randomised controlled trial of social behaviour and network therapy and motivational enhancement therapy in 742 people with alcohol problems who would normally be offered treatment. This paper found that in the six months prior to treatment there was mean healthcare costs of £1,157 and social care costs of £69 across the two arms. Uplifted to 2024 costs and halved to reflect our three-month cycle this represents a combined health and social care cost of £1,223. No cost was attached to the abstinent state as the median cost across both treated groups was zero and we assumed that the proportion of people who were not drinking would have the lowest costs.

Quality of life

Utilities were taken from the UKATT trial [17]. The trial reported a baseline mean of 0.57 for the combined trial population. This was used as the utility for the relapsed state as an inclusion criterion for the trial was to have an alcohol problem sufficient to be offered treatment in the UK. The utility for the abstinent state was taken as the upper confidence interval of the post-treatment utility across both treatment arms (0.67). The upper confidence interval was chosen because the group would include both abstainers and relapsed and it is assumed that the

abstainers would have higher quality of life. The utility on treatment is assumed to be an average of the relapsed and abstainer values (0.62),

Opioid model

Transition probabilities

Relapse rates in the opioid model are taken from Simpson and Marsh [19], who report US cohort data from 1986. Although this is quite dated and may not be directly applicable to the UK population in 2025, it was the most suitable data identified in the form required to populate the model. The impact of using those values is tested in sensitivity analysis. Table 3 of Simpson and Marsh estimates that 35% of people will relapse immediately on completion of treatment. This is taken as the cycle 1 relapse rate. Table 4 of Simpson and Marsh reports that of people achieving abstinence for 3 months, 80% will still be abstinent at 12 months. This equates to a 9-month relapse rate of 20% and a 3-month relapse rate of 6.3% for cycles 2-4. For people still abstinent at 12 months, 16.25% (from Table 4 (80%-67%)/80%) will relapse by 24 months equating to a 3-month relapse rate of 3.8% for cycles 5-8. Table 4 suggests that a further 11.94% ((67%-59%)/67%) will relapse before month 42. This equates to an ongoing 3-month cycle rate of 1.9%.

It is assumed that 90% of people who relapse seek treatment. The recovery rate after treatment is based on national UK statistics for opioid misuse (Adult substance misuse treatment statistics 2023 to 2024: report - GOV.UK) [1]. 23% of people achieve recovery after an average of 3.3 years of treatment. This equates to a three-month rate of 1.6%. No spontaneous recovery is assumed given that such a small proportion of people who relapse achieve abstinence, even with treatment. Mortality rates are taken from Kelty et al, 2018 [20] with people in the medically supervised withdrawal state 6.04 times more likely to die, people off treatment 6.14 times more likely to die and people in the medication for opioid use disorder state 3.55 times more likely to die than population norm mortality rates.

Impact of Subreal App

See alcohol model

Costs

As for alcohol, the costs per day are taken from the commissioning tool table shown in Figure SM1. The proportion of people having each treatment are taken from the national substance misuse data [1]. Days of treatment are retained at 199 in a two-cycle period for consistency with the alcohol model but as people can remain in medication for opioid use disorder state indefinitely, the actual period of treatment for people in the opioid model will be much longer.

Table SM2 - updating and weighting opioid costs (GBP)

Element of treatment – cost per day	2016-2017 (see Fig 1)	2023-2024	Days of treatment	Proportion of people	Total cost
Pharmacological	8.17	10.42	199	0.937	1,942.89
Psychosocial	10.19	13.00	199	0.993	2,568.09
In-patient	164.72	210.08	14	0.032	94.11
Residential	103.57	132.09	30	0.011	43.59
Per 6-month treatment					**4,648.68**
Per 3-month cycle					**2,324.34**

No costs are attached to the relapsed state in the opioid model as the sources are inconsistent and several sources suggest that there is no significant difference between healthcare costs (other than treatment) in populations in the different health states.

Quality of life

The utility value attached to the abstinent state is assumed to be equivalent to the UK population norm from Janssen et al, 2018 for the 35-44-year-old age group. It was not reduced over time in line with the approach adopted in the External Assessment Group's (EAG's) approach in National Institute for Health and Care Excellence Technology Appraisal 115 (TA115) [22]. TA115 concerned oral naltrexone as a treatment for relapse prevention in formerly opioid dependent drug users. The EAG found little data on the quality of life to inform their model so undertook an elicitation exercise with a panel of members of the public attached to their academic institution. They used the standard gamble method to value different health states which were then weighted by the proportion of patients in each of those states in a treatment trial. The resulting weights were 0.84 for patients on treatment and 0.63 for patients not on treatment.
