# Supplementary figures and images for: Prediction of Relapse Using Digital Technology in People in Recovery From Substance Use Disorders: Early Economic Evaluation With a Case Study of the Subreal App
Source: JMIR Form Res. 2026 Apr 14;10:e87186. doi: 10.2196/87186 (PMC13078403; doi:10.2196/87186)

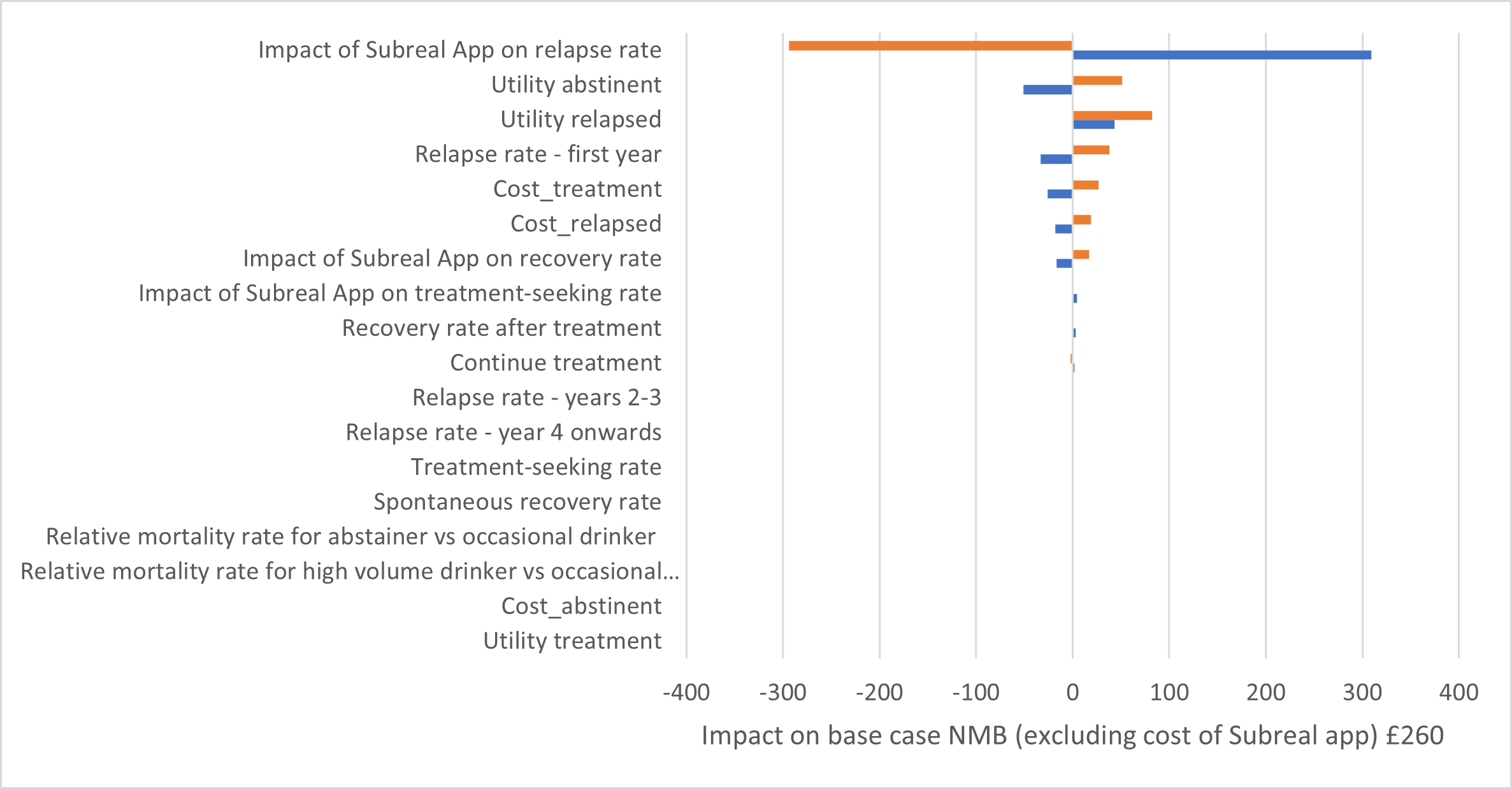

Supplement: Multimedia Appendix 2 [file formative-v10-e87186-s002.png]

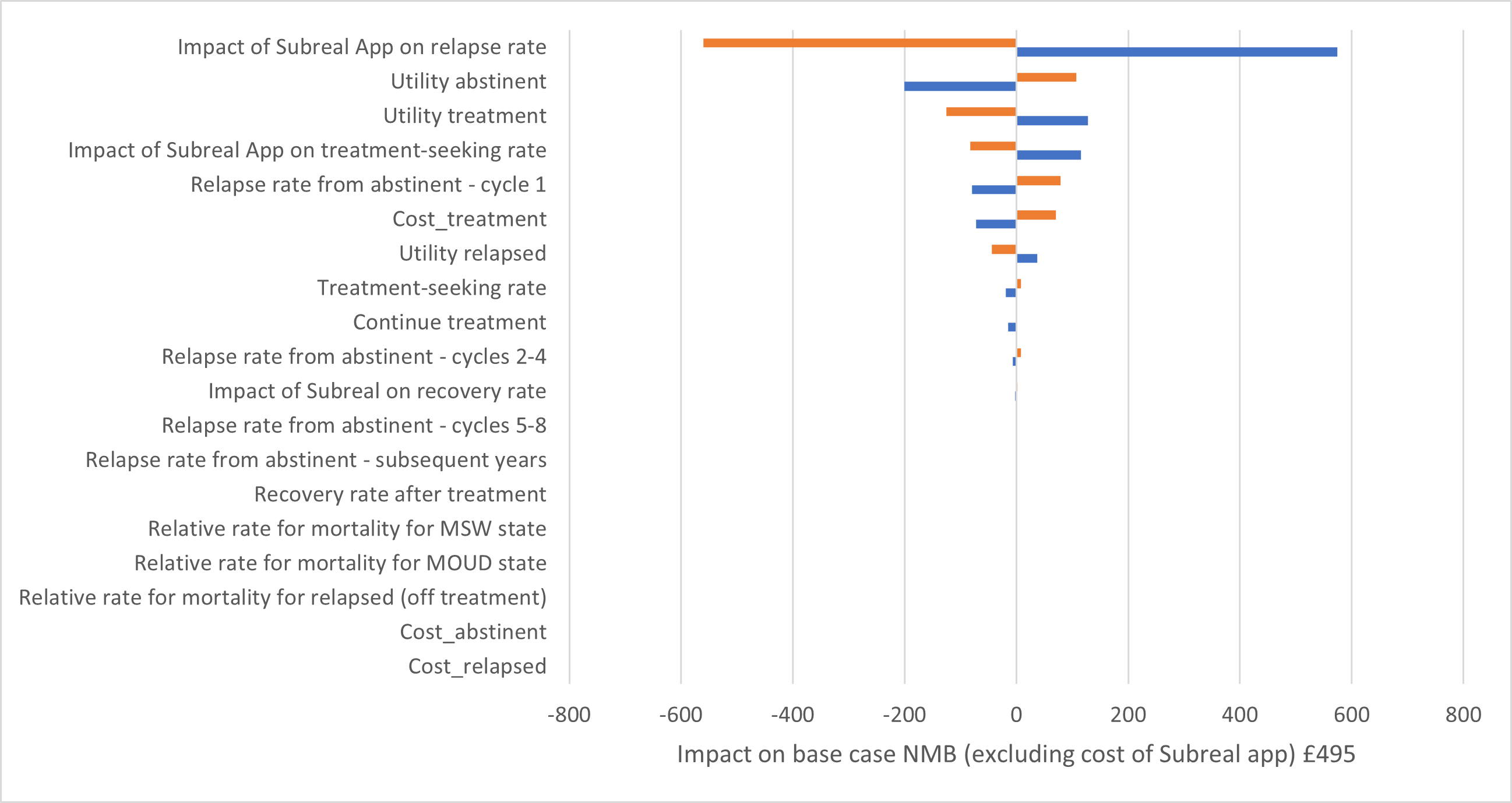

Supplement: Multimedia Appendix 3 [file formative-v10-e87186-s003.png]
