# Supplementary material for: Prediction of Relapse Using Digital Technology in People in Recovery From Substance Use Disorders: Early Economic Evaluation With a Case Study of the Subreal App
Source: JMIR Form Res. 2026 Apr 14;10:e87186. doi: 10.2196/87186 (PMC13078403; doi:10.2196/87186)
Supplement: Checklist 1 [file formative-v10-e87186-s004.pdf]

MMA 1

CHEERS 2022 Checklist

Topic	No.	Item	Location where item is reported
**Title**			
	1	Identify the study as an economic evaluation and specify the interventions being compared.	Title and structured abstract
**Abstract**			
	2	Provide a structured summary that highlights context, key methods, results, and alternative analyses.	Structured abstract
**Introduction**			
**Background and objectives**	3	Give the context for the study, the study question, and its practical relevance for decision making in policy or practice.	Introduction
**Methods**			
**Health economic analysis plan**	4	Indicate whether a health economic analysis plan was developed and where available.	Methods - pre-registration
**Study population**	5	Describe characteristics of the study population (such as age range, demographics, socioeconomic, or clinical characteristics).	Methods - decision problem
**Setting and location**	6	Provide relevant contextual information that may influence findings.	Methods - analytic approach
**Comparators**	7	Describe the interventions or strategies being compared and why chosen.	Methods - decision problem
**Perspective**	8	State the perspective(s) adopted by the study and why chosen.	Methods - analytic approach
**Time horizon**	9	State the time horizon for the study and why appropriate.	Methods - analytic approach
**Discount rate**	10	Report the discount rate(s) and reason chosen.	Methods - analytic approach

Topic	No.	Item	Location where item is reported
**Selection of outcomes**	11	Describe what outcomes were used as the measure(s) of benefit(s) and harm(s).	Methods - analytic appoach
**Measurement of outcomes**	12	Describe how outcomes used to capture benefit(s) and harm(s) were measured.	Methods - parameter estimates and Supplementary Materials
**Valuation of outcomes**	13	Describe the population and methods used to measure and value outcomes.	Supplementary Materials
**Measurement and valuation of resources and costs**	14	Describe how costs were valued.	Supplementary Materials
**Currency, price date, and conversion**	15	Report the dates of the estimated resource quantities and unit costs, plus the currency and year of conversion.	Supplementary Materials
**Rationale and description of model**	16	If modelling is used, describe in detail and why used. Report if the model is publicly available and where it can be accessed.	Methods - structure of the model. Strengths and limitations for availability of model.
**Analytics and assumptions**	17	Describe any methods for analysing or statistically transforming data, any extrapolation methods, and approaches for validating any model used.	Supplementary materials for any calculations of costs and outcomes. Validation discussed in Methods - validation.
**Characterising heterogeneity**	18	Describe any methods used for estimating how the results of the study vary for subgroups.	No subgroup analysis - Methods - Dealing with uncertainty
**Characterising distributional effects**	19	Describe how impacts are distributed across different individuals or adjustments made to reflect priority populations.	No analysis of distributional effects - Methods - Dealing with uncertainty
**Characterising uncertainty**	20	Describe methods to characterise any sources of uncertainty in the analysis.	Methods - dealing with uncertainty
**Approach to engagement with patients and others affected by the study**	21	Describe any approaches to engage patients or service recipients, the general public, communities, or stakeholders (such as clinicians or payers) in the design of the study.	Methods - validation
**Results**			

Topic	No.	Item	Location where item is reported
**Study parameters**	22	Report all analytic inputs (such as values, ranges, references) including uncertainty or distributional assumptions.	Methods - Parameter estimates and Supplementary Materials
**Summary of main results**	23	Report the mean values for the main categories of costs and outcomes of interest and summarise them in the most appropriate overall measure.	Results - Base case results
**Effect of uncertainty**	24	Describe how uncertainty about analytic judgments, inputs, or projections affect findings. Report the effect of choice of discount rate and time horizon, if applicable.	Results - Threshold analysis and One-way sensitivity analysis
**Effect of engagement with patients and others affected by the study**	25	Report on any difference patient/service recipient, general public, community, or stakeholder involvement made to the approach or findings of the study	Not applicable as no stakeholders other than clinician were involved
**Discussion**			
**Study findings, limitations, generalisability, and current knowledge**	26	Report key findings, limitations, ethical or equity considerations not captured, and how these could affect patients, policy, or practice.	Discussion
**Other relevant information**			
**Source of funding**	27	Describe how the study was funded and any role of the funder in the identification, design, conduct, and reporting of the analysis	Title sheet
**Conflicts of interest**	28	Report authors conflicts of interest according to journal or International Committee of Medical Journal Editors requirements.	Title sheet

From: Husereau D, Drummond M, Augustovski F, et al. Consolidated Health Economic Evaluation Reporting Standards 2022 (CHEERS 2022) Explanation and Elaboration: A Report of the ISPOR CHEERS II Good Practices Task Force. Value Health 2022;25. doi:10.1016/j.jval.2021.10.008
